# Three datasets for nutrition environment measures of food outlets located in the Lower Mississippi Delta region of the United States

**DOI:** 10.12688/f1000research.27192.2

**Published:** 2021-05-24

**Authors:** Jessica L. Thomson, Alicia S. Landry

**Affiliations:** 1 Delta Human Nutrition Research Program, USDA Agricultural Research Service, Stoneville, MS, 38776, USA; 2 Department of Family and Consumer Sciences, University of Central Arkansas, Conway, AR, 72035, USA

**Keywords:** Food environment, nutrition, grocery stores, convenience stores, restaurants, Nutrition Environment Measures Survey, Lower Mississippi Delta, rural

## Abstract

This data note provides details of a research database containing 266 food outlets located in five rural towns in the Lower Mississippi Delta region of Mississippi, whose nutrition environments were measured from 2016 to 2018.  The food outlet types include grocery stores, convenience stores, full-service restaurants, and fast food restaurants.  The purpose of this publication is to describe the three datasets for external researchers who may be interested in making use of them.  The datasets are available from the USDA National Agricultural Library’s Ag Data Commons under a CC0 1.0 Universal License: https://doi.org/10.15482/USDA.ADC/1503704.

## Introduction

The Mississippi River Delta region is among the most socioeconomically disadvantaged areas of the United States (US) with less healthful food environments (e.g., low access to healthful foods, food insecurity) and poorer health outcomes than non-Delta counties in the same states and the nation ^1^. Accessibility (location of healthful food outlets near neighborhoods, particularly in low-income and rural areas), availability (healthful options in local food outlets), and affordability (reasonable prices) of nutrient-dense foods are crucial to facilitate adoption of a healthful diet ^2– 4^. To inform future nutrition interventions designed for residents of the Lower Mississippi, the Delta Food Outlets Study was conducted to measure nutrition environments of towns located in this region. 

## Methods

Delta Food Outlets was an observational study designed to collect data on food outlets located in five rural Lower Mississippi Delta towns. These towns were selected because participants of a previously conducted nutrition intervention resided in the five towns and assessing environmental exposures potentially influencing their dietary habits was of interest to researchers ^5^. The population of the five towns ranged from 1,750 to 32,612 residents. The percentage of the towns’population that was African American ranged from 49% to 91% and the percentage that lived below the federal poverty level ranged from 29% to 51%. Food outlet types included grocery stores, convenience stores, full-service restaurants, and fast food restaurants. The study was approved and classified as exempt by the Institutional Review Board of Delta State University. Data collection occurred from March 2016 through September 2018.

Grocery stores were identified by referencing two sources – the US Department of Agriculture (USDA) Food and Nutrition Service Supplemental Nutrition Assistance Program (SNAP) retailer locator ^6^ and the Mississippi State Department of Health Restaurant and Food Facility Inspections website ^7^. Convenience stores were identified by referencing three sources – the SNAP retailer locator ^6^, the B2B Yellow Pages website ^8^, and lists of current privilege licenses obtained from city clerks. Restaurants were identified by referencing the Mississippi State Department of Health Restaurant and Food Facility Inspections website ^7^. Food outlets were classified using operational definitions contained in the Economic Research Service’s Food Environment Atlas documentation ^9^. Grocery stores were defined as supermarkets and smaller grocery stores primarily engaged in retailing a general line of food, such as canned and frozen foods; fresh fruits and vegetables; and fresh and prepared meats, fish, and poultry. Convenience stores were defined as stores primarily engaged in retailing a limited line of goods that generally includes milk, bread, soda, and snacks. Full-service restaurants were defined as restaurants that provide food service to patrons who order and are served while seated and pay after eating. Fast food restaurants were defined as restaurants that provide food services (excluding snack and nonalcoholic beverage bars) where patrons generally order or select items and pay before eating. Before measurement, all outlets were physically visited to ensure that they were open, sold food, and were classified correctly. While the 266 food outlets included in the datasets represent the entire population of these types of food outlets in the five towns, they may not be representative of all such outlets located in rural Lower Mississippi Delta towns.

Nutrition environments of the food outlets were measured using the Nutrition Environment Measures Survey (NEMS) for grocery stores (NEMS-S), convenience stores (NEMS-CS), and restaurants (NEMS-R) ^10^. NEMS tools are validated observational measures of retail store nutrition environments that focus on the availability of healthful food choices, quality of fresh produce (acceptable or unacceptable based on visual inspection), and comparative pricing between healthful and less healthful options (e.g., lower vs. higher fat, no added vs. added sugars, whole grain vs. refined grain) in 11 common categories ^11^. The categories included milk, fruits, vegetables, ground beef, hot dogs, frozen dinners, baked goods, beverages, bread, chips, and cereal. For the restaurants, main dishes and main dish salads were measured rather than ground beef, hot dogs, and frozen dinners, and baked goods and cereal were not measured. Additionally, facilitators (e.g., nutrition information available) and barriers (e.g., supersizing available) for healthful eating were measured in restaurants. A comprehensive description of the Delta Food Outlets Study methodology and measures has been published elsewhere ^5^.

The NEMS tools were recreated as electronic surveys using Snap Surveys software (version 11.20, Snap Surveys Ltd). All data were collected via tablets loaded with Snap Surveys software and stored on the Snap WebHost, an online mobile and secure survey management system. For quality assurance purposes, 25% of the food outlets were randomly selected for duplicate measurement. Discrepancies between measurements were discussed between senior researchers and data collectors and resolved (e.g., outlet re-visited to determine correct value).

Food outlets were scored using algorithms provided for the NEMS tools. Higher scores indicate a more healthful nutrition environment. The datasets include raw scores for each item, component scores (i.e., food groups, availability, pricing, quality, healthy options, facilitators, and barriers), and total scores. Scoring was performed using SAS® (version 9.4, SAS Institute Inc).

## Data availability

USDA National Agricultural Library’s Ag Data Commons: Delta Food Outlets Study, https://doi.org/10.15482/USDA.ADC/1503704 ^12^.

This project contains all three datasets – NEMS-C (convenience stores), NEMS-G (grocery stores), and NEMS-R (restaurants) – along with their corresponding data dictionaries.

Data are available under the terms of the Creative Commons Zero "No rights reserved" data waiver (CC0 1.0 Public domain dedication).

## References

[1] Gennuso KP Jovaag A Catlin BB: Assessment of Factors Contributing to Health Outcomes in the Eight States of the Mississippi Delta Region. *Prev Chronic Dis.* 2016;13(3):E33. 10.5888/pcd13.150440 26940300 PMC4778371

[2] Cohen B Andrews M Kantor LS: Community Food Security Assessment Toolkit.2002. Reference Source

[3] Laraia B Siega-Riz AM Kaufman JS: Proximity of supermarkets is positively associated with diet quality index for pregnancy. *Prev Med.* 2004;39(5):869–875. 10.1016/j.ypmed.2004.03.018 15475018

[4] Rose D Richards R: Food store access and household fruit and vegetable use among participants in the US Food Stamp Program. *Public Health Nutr.* 2004;7(8):1081–1088. 10.1079/PHN2004648 15548347

[5] Thomson JL Goodman MH Landry AS: Measurement of Nutrition Environments in Grocery Stores, Convenience Stores, and Restaurants in the Lower Mississippi Delta. *Prev Chronic Dis.* 2020;17:E24. 10.5888/pcd17.190293 32163354 PMC7085910

[6] US Department of Agriculture Food and Nutrition Service: Where can I use SNAP EBT?Accessed March 15, 2016. Reference Source

[7] Mississippi State Department of Health: Restaurant and Food Facility Inspections. Accessed March 15, 2016. Reference Source

[8] b2bBiz.com: B2BYellowpages.com. Accessed March 15, 2016. Reference Source

[9] US Department of Agriculture Economic Research Service: Food Environment Atlas. Accessed August 8, 2019. Reference Source

[10] The University of Pennsylvania: Nutrition Environment Measures Survey. Accessed May 23, 2018. Reference Source

[11] Glanz K Sallis JF Saelens BE: Nutrition Environment Measures Survey in stores (NEMS-S): development and evaluation. *Am J Prev Med.* 2007;32(4):282–289. 10.1016/j.amepre.2006.12.019 17383559

[12] Thomson JL: Delta Food Outlets Study. Ag Data Commons. Accessed 2020-10-19. 10.15482/USDA.ADC/1503704

