# Supplementary material for: Three datasets for nutrition environment measures of food outlets located in the Lower Mississippi Delta region of the United States
Source: F1000Res. 2021 May 24;9:1307. Originally published 2020 Nov 9. [Version 2] doi: 10.12688/f1000research.27192.2 (PMC8129811; doi:10.12688/f1000research.27192.2)
Supplement: Peer review history

# Peer review: Reviewer response for version 2

**DOI:** 10.5256/f1000research.56999.r85979

**Published:** 2021-05-24

**Authors:** Donald Diego Rose

**Affiliations:** 1 School of Public Health & Tropical Medicine, Tulane University, New Orleans, LA, USA

All looks good. No further comments.

Are sufficient details of methods and materials provided to allow replication by others?

Partly

Is the rationale for creating the dataset(s) clearly described?

Yes

Are the datasets clearly presented in a useable and accessible format?

Partly

Are the protocols appropriate and is the work technically sound?

Yes

Reviewer Expertise:

Public health nutrition, food security, food environment

I confirm that I have read this submission and believe that I have an appropriate level of expertise to confirm that it is of an acceptable scientific standard.

# Peer review: Reviewer response for version 1

**DOI:** 10.5256/f1000research.30043.r83927

**Published:** 2021-05-17

**Authors:** Caitlin Caspi

**Affiliations:** 1 Rudd Center for Food Policy and Obesity, University of Connecticut, Hartford, CT, USA

This a useful, detailed, and fairly recent data set that can be used by researchers to study the food environment in the Lower Mississippi Delta region. The data note concisely describes the data and is likely to be helpful documentation. 

 There were some places where more detail would have been useful.

1. What are the characteristics of the towns of interest? Adding in the range of population size of the towns would be helpful. Were there large metropolitan areas that were excluded because this study was intentionally focused on towns? And/or can the authors make any statement in the note about how the towns may not be representative of the larger Lower Mississippi Delta area food environment? Some information like this would be helpful for readers in determining if the dataset is appropriate for use for addressing their research question.
2. Classifying food retail outlets (i.e., grocery stores vs. convenience stores, restaurants vs. fast food outlets) can be challenging. Potential misclassification of store types is a major topic of discussion in food environment databases. Consider adding in more detail about how food stores were classified. The definitions used for the food retail outlet categories would be helpful (I see they are defined in the longer paper), but even more helpful would be a description of how decisions were made about how to classify a food outlet (e.g., based on one of the food lists, and then verified during data collection in person?).
3. Relatedly, the authors reference the documentation for the USDA Food Environment Atlas for how stores were classified. However, the link goes to the Atlas, not the documentation, so it’s hard to find more information on how stores were classified. It would be helpful to have a direct link.

Are sufficient details of methods and materials provided to allow replication by others?

Partly

Is the rationale for creating the dataset(s) clearly described?

Yes

Are the datasets clearly presented in a useable and accessible format?

Yes

Are the protocols appropriate and is the work technically sound?

Yes

Reviewer Expertise:

Food environment, food security, policies and interventions to address diet-related behaviors

I confirm that I have read this submission and believe that I have an appropriate level of expertise to confirm that it is of an acceptable scientific standard.

# Response

**Published:** 2021-05-18

**Authors:** Jessica Thomson

**Affiliations:** USDA Agricultural Research Service, USA

We thank Dr. Caspi for their thoughtful and thorough review of our manuscript. We have incorporated Dr. Caspi’s suggestions for strengthening our Data Note about the Delta Food Outlets datasets. Our responses to Dr. Caspi’s comments follow.

 Pertaining to the towns: What are the characteristics of the towns of interest? Adding in the range of population size of the towns would be helpful. Were there large metropolitan areas that were excluded because this study was intentionally focused on towns? And/or can the authors make any statement in the note about how the towns may not be representative of the larger Lower Mississippi Delta area food environment? Some information like this would be helpful for readers in determining if the dataset is appropriate for use for addressing their research question.

**Response: We have added characteristics of the 5 towns including ranges for population size, percentage of African American residents, and percentage of residents living below the federal poverty level. Additionally, we added our reason for selecting these 5 towns – because participants of a previously conducted nutrition intervention resided in the 5 towns and assessing environmental exposures potentially influencing their dietary habits was of interest. We chose not to add a note about the non-representative nature of the towns because we felt it was redundant with our comment about the non-representative nature of the food outlets.**

 Classifying food retail outlets (i.e., grocery stores vs. convenience stores, restaurants vs. fast food outlets) can be challenging. Potential misclassification of store types is a major topic of discussion in food environment databases. Consider adding in more detail about how food stores were classified. The definitions used for the food retail outlet categories would be helpful (I see they are defined in the longer paper), but even more helpful would be a description of how decisions were made about how to classify a food outlet (e.g., based on one of the food lists, and then verified during data collection in person?). **Response: We have added more detail about definitions used to classify food outlets. Additionally, we added that before measurement, all outlets were physically visited to ensure that they were open, sold food, and were classified correctly.**

 Relatedly, the authors reference the documentation for the USDA Food Environment Atlas for how stores were classified. However, the link goes to the Atlas, not the documentation, so it’s hard to find more information on how stores were classified. It would be helpful to have a direct link.

**Response: We have updated the Food Environment Atlas reference to include a direct link to the documentation.**

# Peer review: Reviewer response for version 1

**DOI:** 10.5256/f1000research.30043.r74475

**Published:** 2020-12-22

**Authors:** Donald Diego Rose

**Affiliations:** 1 School of Public Health & Tropical Medicine, Tulane University, New Orleans, LA, USA

Congratulations on a great piece of primary research on the food environment in the lower Mississippi Delta! I think it is great that you are listing the datasets in a way that can be found by other researchers, and the Data Note through F1000Resaerch is a great vehicle for this. I have just a few comments to strengthen the Note for potential users. See below for some minor changes to be made:

1. “located in five rural Lower Mississippi Delta towns of interest to researchers”
  I think it would be useful to explain why or how these towns were chosen. Why were they of interest to the researchers? Are they representative of a specific region? Was it just a convenience sample? Do they provide a range of geographies or socio-economic conditions? I realize this is probably in the source article (#5), but a couple of sentences here answering one or more of these questions to orient potential users of the data would be helpful.
  Also, can you list the names of the towns? If there is a confidentiality issue, I would understand. But if not, it would be helpful for potential users of the data to know which towns.
2. “that focus on the availability of healthful food choices, quality of fresh produce, and comparative pricing between healthful and less healthful options in 11 common categories”
  I know this is well documented in other places, but it would be useful in this Data Note to list one example of each of these and to also list all 11 food categories. You could add parenthetical comments, for example:
*availability of healthful food choices (e.g, non-fat milk or whole grain bread, etc), quality of fresh produce (as scored on a X-point scale), and…11 common categories. The categories include a, b, c, …. *
  Then list them all. It’s easy to do and gives the reader a better sense of the data.
3. “Discrepancies between measurements were discussed and resolved.”
  Discussed by whom? The investigators? Graduate student assistants? Just make clear this clear.
4. “The higher the ratio score, the more healthful the nutrition environment.”
  Can you indicate if the raw scores and the component scores are also available on the database? Either way is fine, but this would be helpful to know because some feel that the overall NEMS measures are like black box numbers that may not be helpful for a specific campaign to increase fruits and vegetables. See, for example, the references below that allow assessment for targeted campaigns to increase fruits and vegetables or decrease empty calorie snack foods. You may wish to include one or both of these references.
  Farley TA, Rice JC, Bodor JN, Cohen DA, Blumenthal RN, Rose D. "Measuring the Food Environment: Shelf Space of Fruits, Vegetables, and Snack Foods in Stores," *Journal of Urban Health*, 2009;86:672-682. ^1^
  Miller C, Bodor JN, Rose D. "Measuring the food environment: A systematic technique for characterizing food stores using display counts," *Journal of Environmental and Public Health* 2012; doi:10.1155/2012/707860. ^2^

Are sufficient details of methods and materials provided to allow replication by others?

Partly

Is the rationale for creating the dataset(s) clearly described?

Yes

Are the datasets clearly presented in a useable and accessible format?

Partly

Are the protocols appropriate and is the work technically sound?

Yes

Reviewer Expertise:

Public health nutrition, food security, food environment

I confirm that I have read this submission and believe that I have an appropriate level of expertise to confirm that it is of an acceptable scientific standard.

## References

[1] : Measuring the food environment: shelf space of fruits, vegetables, and snack foods in stores. *J Urban Health*.2009;86(5) : 10.1007/s11524-009-9390-3 672-82 10.1007/s11524-009-9390-3 19603271PMC2729874

[2] : Measuring the food environment: a systematic technique for characterizing food stores using display counts. *J Environ Public Health*.2012;2012: 10.1155/2012/707860 707860 10.1155/2012/707860 22701497PMC3373162

# Response

**Published:** 2021-05-18

**Authors:** Jessica Thomson

**Affiliations:** USDA Agricultural Research Service, USA

We thank Dr. Rose for their thoughtful and thorough review of our manuscript. We have incorporated Dr. Rose’s suggestions for strengthening our Data Note about the Delta Food Outlets datasets. Our responses to Dr. Rose’s comments follow.

 Pertaining to the towns: I think it would be useful to explain why or how these towns were chosen. Why were they of interest to the researchers? Are they representative of a specific region? Was it just a convenience sample? Do they provide a range of geographies or socio-economic conditions? I realize this is probably in the source article (#5), but a couple of sentences here answering one or more of these questions to orient potential users of the data would be helpful. Also, can you list the names of the towns? If there is a confidentiality issue, I would understand. But if not, it would be helpful for potential users of the data to know which towns.

**Response: We have added our reason for selecting these 5 towns – because participants of a previously conducted nutrition intervention resided in the 5 towns and assessing environmental exposures potentially influencing their dietary habits was of interest. We have chosen not to name the 5 towns due to confidentiality issues.**

 Pertaining to the NEMS instruments: I know this is well documented in other places, but it would be useful in this Data Note to list one example of each of these and to also list all 11 food categories. You could add parenthetical comments, for example: *availability of healthful food choices (e.g, non-fat milk or whole grain bread, etc), quality of fresh produce (as scored on a X-point scale), and…11 common categories. The categories include a, b, c,..*Then list them all. It’s easy to do and gives the reader a better sense of the data.

**Response: We have added examples of the measures’ focus and listed the 11 common categories as suggested.**

 Pertaining to measurement discrepancies: Discussed by whom? The investigators? Graduate student assistants? Just make clear this clear.

**Response: We have clarified that discrepancies were discussed between senior researchers and data collectors and resolved (e.g., outlet re-visited to determine correct value).**

 Pertaining to the scores: Can you indicate if the raw scores and the component scores are also available on the database? Either way is fine, but this would be helpful to know because some feel that the overall NEMS measures are like black box numbers that may not be helpful for a specific campaign to increase fruits and vegetables. See, for example, the references below that allow assessment for targeted campaigns to increase fruits and vegetables or decrease empty calorie snack foods. You may wish to include one or both of these references. Farley TA, Rice JC, Bodor JN, Cohen DA, Blumenthal RN, Rose D. "Measuring the Food Environment: Shelf Space of Fruits, Vegetables, and Snack Foods in Stores," Journal of Urban Health, 2009;86:672-682.1. Miller C, Bodor JN, Rose D. "Measuring the food environment: A systematic technique for characterizing food stores using display counts," Journal of Environmental and Public Health 2012; doi:10.1155/2012/707860.

**Response: We apologize for this oversight in describing our datasets. Raw scores, component scores, and total scores are available in the datasets. In fact, we did not include ratio scores in the datasets because they were created specifically for the purposes of our study and may not be relevant or useful for other researchers. Hence, we have added text about the availability of raw, component, and total scores and removed the text about ratio scores. While the references provided are very interesting, we did not feel it was necessary to include them in our Data Note given the availability of raw scores in the datasets.**
